# Forward genetic approach identifies a phylogenetically conserved serine residue critical for the catalytic activity of UBIQUITIN-SPECIFIC PROTEASE 12 in Arabidopsis

**DOI:** 10.1038/s41598-024-77232-w

**Published:** 2024-10-25

**Authors:** Anita Hajdu, Dóra Vivien Nyári, Éva Ádám, Yeon Jeong Kim, David E. Somers, Dániel Silhavy, Ferenc Nagy, László Kozma-Bognár

**Affiliations:** 1 Department of Genetics, Faculty of Sciences and Informatics, University of Szeged, Szeged, H- 6726 Hungary; 2 Institute of Plant Biology, Biological Research Centre, Hungarian Research Network (HUN-REN), Szeged, H-6726 Hungary; 3 Department of Medical Genetics, Faculty of Medicine, University of Szeged, Szeged, H-6720 Hungary; 4 Doctoral School in Biology, Faculty of Science and Informatics, University of Szeged, Szeged, H-6726 Hungary; 5 Department of Molecular Genetics, Ohio State University, Columbus, OH USA

**Keywords:** Ubiquitin proteases, UBP12, De-ubiquitination, Circadian clock, Flowering time, Phosphorylation, Proteases, Plant signalling, Circadian rhythms in plants, Proteolysis in plants, Gene regulation, Deubiquitylating enzymes

## Abstract

Circadian clocks rely on transcriptional/translational feedback loops involving clock genes and their corresponding proteins. While the primary oscillations originate from gene expression, the precise control of clock protein stability plays a pivotal role in establishing the 24-hour circadian rhythms. Most clock proteins are degraded through the ubiquitin/26S proteasome pathway, yet the enzymes responsible for ubiquitination and deubiquitination remain poorly characterised. We identified a missense allele (*ubp12-3*, S327F) of the UBP12 gene/protein in Arabidopsis. Despite *ubp12-3* exhibited a short period phenotype similar to that of a loss-of-function allele, molecular analysis indicated elevated protease activity in *ubp12-3*. We demonstrated that early flowering of *ubp12* mutants is a result of the shortened circadian period rather than a direct alteration of UBP12 function. Analysis of protease activity of non-phosphorylatable (S327A, S327F) and phosphomimetic (S327D) derivatives in bacteria suggested that phosphorylation of serine 327 inhibits UBP12 enzymatic activity, which could explain the over-functioning of S327F in vivo. We showed that phosphomimetic mutations of the conserved serine in the *Neurospora* and human orthologues reduced ubiquitin cleavage activity suggesting that not only the primary structures of UBP12-like enzymes are phylogenetically conserved across a wide range of species, but also the molecular mechanisms governing their enzymatic activity.

**Supplementary Information:**

The online version contains supplementary material available at 10.1038/s41598-024-77232-w.

## Introduction

Eukaryotic circadian clocks are oscillating gene networks, where clock genes and the encoded clock proteins mutually regulate the expression of each other in a self-sustained cyclic manner with app. 24 h periods. The primary oscillation is generated at the level of clock gene transcription and is relayed to the rhythmic, daily regulation of a significant portion of the genome. The coordinated temporal regulation of these gene sets provides the basis of scheduling vital molecular and physiological processes to the most appropriate time of the day, thus fulfilling the biological function of circadian clocks. In order to maintain correct timing, circadian oscillators are synchronised (entrained) to the environmental day/night cycles on a daily basis. One of the most effective environmental signals for entrainment is light. Light signals not only set the phase of the clock, but also modulate the pace of the oscillation in a fluence-rate dependent manner^1^.

The plant circadian oscillator - similar to the clocks in other model species from fungi to mammals - is composed of several interlocked transcriptional/translational regulatory loops. The first discovered component of the plant clock, CIRCADIAN CLOCK ASSOCIATED1 (CCA1) and its close homolog LATE ELONGATED HYPOCOTYL (LHY) are MYB-related transcription factors showing the peak of their expression in the morning^2^. Later during the day and most of the night, transcription of *CCA1/LHY* is repressed by the sequentially expressed members of the PSEUDO RESPONSE-REGULATOR (PRR) gene/protein family in the order of PRR9, PRR7, PRR5, and lastly PRR1^3^. PRR1 is also known as TIMING OF CAB EXPRESSION 1 (TOC1) and was the second identified component of the plant clock. As TOC1 protein levels drop around the end of the night, *CCA1/LHY* transcription is re-initiated and reaches the maxima in a few hours after dawn. In turn, CCA1 and LHY repress *PRR* gene expression, thereby closing the central feedback loop^4^. CCA1/LHY as well as TOC1 inhibit the transcription of *LUX ARRHYTMO* (*LUX*) and *EARLY FLOWERING 4* (*ELF4*)^4,5^. The LUX and ELF4 proteins together with the gene product of the early night expressed *ELF3* gene constitute the so-called Evening Complex (EC)^6^. The EC represses expression of *PRR7* and *PRR9* genes, thus promoting *CCA1/LHY* transcription indirectly and forming another regulatory loop^7^. The positive arm of the oscillator is represented by the Myb-like transcription factors NIGHT LIGHT-INDUCIBLE AND CLOCK-REGULATED1 and 2 (LNK1 and 2) and REVEILLE8 (RVE8). The complex of these three proteins directly activates the transcription of *PRR* (*PRR5* and *TOC1*) and *EC* (*LUX* and *ELF4*) genes^8,9^. In turn, TOC1 and other PRRs inhibit the expression of *RVE8* adding one more feedback loop to the clock gene network in plants^8^.

Although the primary oscillation is generated at the transcriptional level, the actual period of the clock is strongly influenced by the turnover of the clock proteins. Ubiquitination and subsequent degradation by the 26 S proteasome is the general way of elimination of clock proteins. F-box proteins functioning as adapters between the ubiquitin ligase complex and the target proteins play an important role in this process. ZEITLUPE (ZTL), the founder member of the ZTL F-box protein family facilitates ubiquitination of TOC1 and PRR5 by the SKP1-like Cul1 F-box (SCF) ubiquitin ligase^10,11^. The stability of ZTL itself is also regulated by ubiquitination. *ZTL* is transcribed constitutively, but association of the ZTL protein with the rhythmically produced GIGANTEA (GI) protein results in the stabilization/maturation and accumulation of ZTL in the evening^12,13^. GI directly interacts with UBIQUITIN-SPECIFIC PROTEASE12 and 13 (UBP12/13) and recruits these enzymes to the GI-ZTL complex to remove ubiquitin moieties and stabilize both GI and ZTL proteins^14^. Consistent with this, both GI and ZTL protein levels are reduced in the *ubp12* mutant displaying a short period circadian phenotype^14,15^. Despite the partial loss of ZTL function, TOC1 protein levels are also decreased in the *ubp12* mutant that corresponds well to the short period phenotype, though. Nevertheless, a similar decline in TOC1 levels was detected in *gi* mutants showing significantly lowered ZTL protein levels^12^. This indicates that changes in TOC1 protein levels could not be simply predicted from alterations in ZTL abundance.

UBP12/13 were also reported to affect photoperiodic flowering in a CONSTANS (CO)-dependent manner^15^. *ubp12* mutants flower markedly earlier than wild-type plants, particularly in short day conditions^15^. Accelerated flowering was explained by the earlier phase of *CO* expression resulting in significantly higher *FLOWERING LOCUS T* (*FT*) mRNA levels in the mutant^15^. Note, that other reports also suggested a CO-independent effect of UBP12/13 on the transcription of key flowering time genes^16^.

UBP12/13 target several functionally not related regulatory proteins for de-ubiquitination. By modulating the protein level of these master regulators, UBP12/13 – in addition to regulating the clock – fine-tune the activity of different hormonal (jasmonate, salicylic acid, brassinosteroid and abscisic acid) or light (blue or ultraviolet light, red/far-red ratio) controlled signalling pathways^17^.

The transcription factor MYC2 is a key regulator of jasmonate-responsive gene expression^18^. UBP12/13 have been shown to stabilize MYC2 proteins by removing the polyubiquitin chains^19^. Therefore, *ubp12/13* mutants accumulate lower levels of MYC2 compared to wild type that results in hyposensitivity to jasmonate^19^.

The stability of most, but not all targets of UBP12/13 are affected by these proteases. Monoubiquitination of histone 2 A (H2Aub1) by the Polycomb repressive complex 1 (PRC1) does not affect the degradation of H2A, but serves as an epigenetic mark recruiting the PRC2, which promotes trimethylation of lysine 27 of histone 3 (H3K27me3) to repress gene expression. Interestingly, this methylation pattern is then stabilized by removing H2Aub1 marks by UBP12/13^20^. As a result, a particular set of PRC2-dependent genes show elevated expression (de-repression) in *ubp12/13* mutants^16,20^.

Here, we report the isolation and characterisation of a novel missense allele of the Arabidopsis *UBP12* gene (*ubp12-3*). The mutation lies within the protease domain of the protein and results in a serine-to-phenylalanine change at a position (S327F), which is remarkably conserved among UBP12-like orthologs from a wide range of taxa, but is missing from other members of the UBP family in Arabidopsis. The *ubp12-3* mutant shows a short period circadian phenotype indistinguishable from that of the *ubp12-1* null mutant. However, analysis of MYC2- and H2A(ub1)-dependent gene expression indicated a generally increased ubiquitin protease activity in the mutant, suggesting that *ubp12-3* is a hypermorphic allele. Interestingly, both *ubp12-1* and *ubp12-3* display accelerated flowering in short day conditions. The flowering phenotypes were rescued by the application of T21-cycles matching the short period of the mutants. This finding demonstrates that the flowering phenotypes were caused primarily by the short period clock phenotype shared by both *ubp* alleles and not directly by the altered or loss of function of UBP12.

In vitro protease assays revealed no differences between the activities of the WT and the S327F mutant UBP12 proteins demonstrating that the mutation per se did not altered the enzymatic activity of UBP12. Nevertheless, testing the activity of phosphomimetic variants S327A (constitutively unphosphorylated) and S327D (constitutively phosphorylated) suggested that phosphorylation of S327 may attenuate the protease activity of UBP12. Based on our findings, we propose a hypothetical model in which phospho-modification fine-tunes the ubiquitin cleavage activity of UBP12. In WT plants a certain proportion of UBP12 could be phosphorylated at S327, and thus inactivated. In the S327F mutant, however, the entire pool of UBP12 should be unphosphorylated and active. This may result in an apparent increase in the overall UBP12-related ubiquitin protease activity in the mutant plants.

## Results

### Identification of a novel short period circadian clock mutant in *Arabidopsis thaliana*

**Fig. 1:**
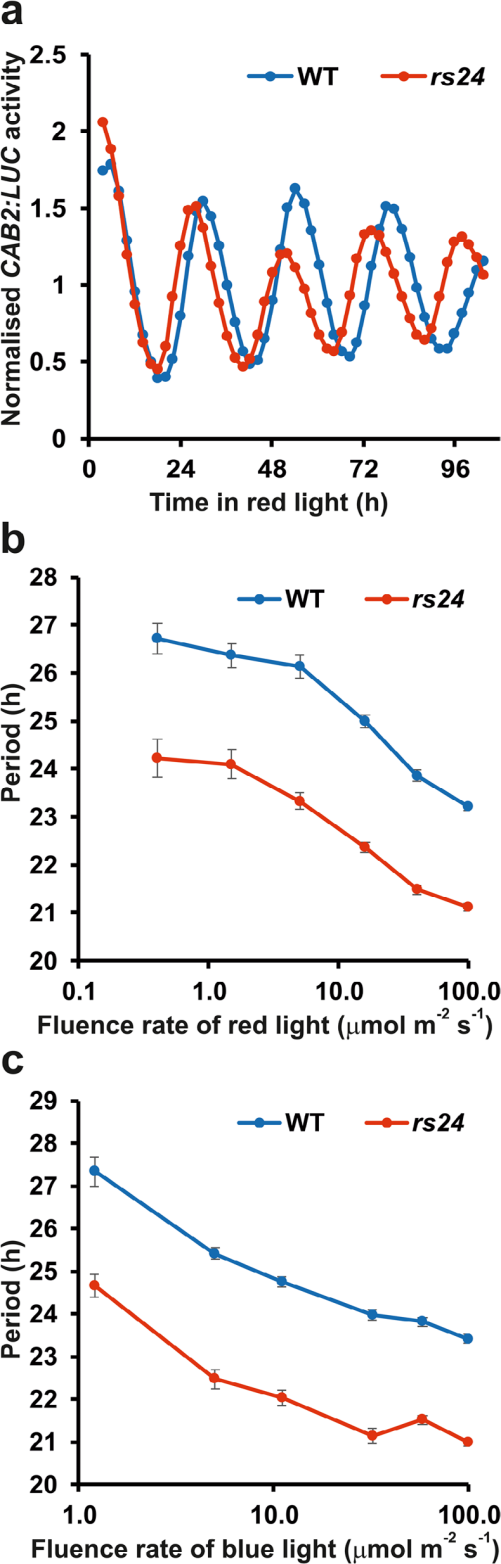
The short period phenotype of *rs24* is independent of the quality and quantity of light. (**a**) Rhythmic traces of *CAB2:LUC* expression in the *rs24* mutant and the corresponding wild type (WT) in continuous red light (10 µmole m^-2^ s^-1^). The graphs represent the averages of 24 seedlings for each genotype. (**b**) Fluence rate response curve for period in continuous red light or (**C**) blue light. WT and *rs24* plants harbouring the *CAB2:LUC* marker were grown in 12 h light:12 h dark photocycles for 7 days and then transferred to continuous red (**B**) or blue (**C**) light at the indicated fluence rates. Rhythmic luminescence was monitored for 4-5 days. Periods were estimated and plotted against the corresponding fluence value. Error bars represent SE values of 3 to 4 independent assays.

An EMS-mutagenized population of a transgenic line expressing the *CHLOROPHYLL A/B-BINDING PROTEIN* (*CAB2*, also known as *LHCB1.1*):*LUC* reporter in the Wassilewskija-2 (Ws-2) accession^21^ was screened in continuous red light for novel circadian mutants^22^. Among others, *red screen 24* (*rs24*) has been identified as a mutant with free-running periods markedly shorter than that of the wild type (Fig. 1a).

Since light has a strong effect on the speed of circadian clocks in general, the period phenotype of *rs24* could be caused by the altered function of the light input pathway to the circadian oscillator. However, in this case the phenotype is expected to be dependent on the quantity and/or quality of the ambient light. To test this, *CAB2:LUC* periods were determined in wild-type and *rs24* plants at different fluences of continuous red (Fig. 1b) or blue (Fig. 1c) light. Essentially the same period differences were detected between wild-type and *rs24* plants over the entire fluence rates tested (Fig. 1b-c). The light quantity- and quality-independent nature of the phenotype suggested that the mutation does not affect the light input to the clock, but probably influences the function of one or more component(s) of the core oscillator.

### *rs24* is a novel missense allele of *UBIQUITIN-SPECIFIC PROTEASE 12* (*UBP12*), which shows a short period phenotype similar to that of the *ubp12-1* null mutant

**Fig. 2:**
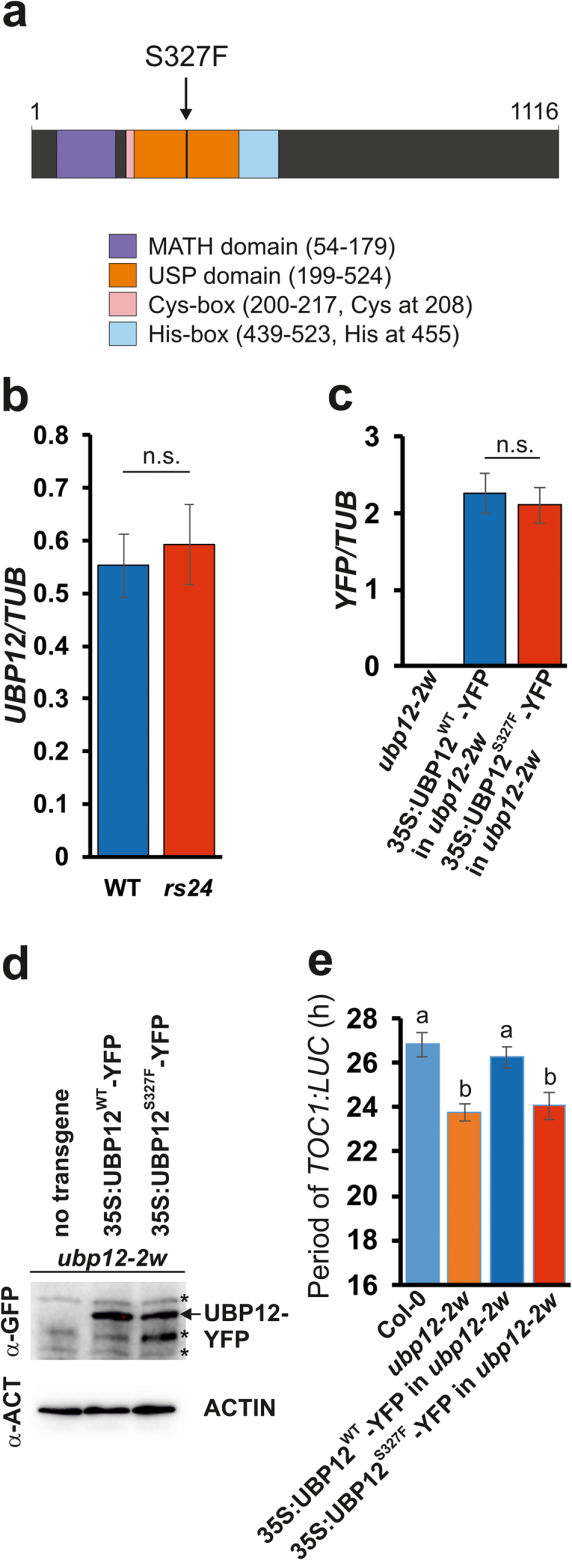
*rs24* is a novel missense allele of the *UBIQUITIN-SPECIFIC PROTEASE 12* (*UBP12*) gene. (**a**) Protein domain structure of UBP12. UBP12 contains a conserved Meprin And TRAF Homology (MATH) domain and a Ubiquitin-Specific Protease (USP) domain in the N-terminus. The USP has conserved cysteine protease enzymatic regions: Cysteine Box (Cys-box) and Histidine Box (His-box). Mutations of the conserved cysteine or histidine residues have been shown to disrupt the deubiquitinating activities of UBP enzymes. The numbers represent the position of landmark amino acid residues. The position of the serine (position 327) residue that is substituted with phenylalanine in *rs24* is indicated by an arrow. (**b**) *UBP12* mRNA levels in WT and *rs24* plants were determined by qPCR assays and normalised to *TUBULIN2/3* (*TUB*) levels. n.s.: not significant as determined by Student's *t* test (*P* > 0.05). (**c**) Wild type UBP12-YFP fusion proteins (UBP12^WT^-YFP) or UBP12-YFP fusion proteins carrying the S327F substitution (UBP12^S327F^-YFP) were expressed from the *35S* promoter in the *ubp12-2w* background carrying the *TOC1:LUC* reporter. A pair of lines showing nearly identical levels of *UBP12-YFP* mRNA were selected (**c**) to test and compare UBP12^WT^-YFP and UBP12^S327F^ protein levels (**d**). Asterisks indicate nonspecific signals present in the non-transformed *ubp12-2w* control as well. Uncropped blots are shown in Supplementary Information 1a. (**e**) *TOC1:LUC* rhythms were monitored in plants expressing UBP12^WT^-YFP and UBP12^S327F^-YFP fusion proteins (same lines shown in panels **C** and **D**) and the corresponding controls indicated. Free running periods in continuous red light (10 µmole m^-2^ s^-1^) conditions were determined and are plotted here. Error bars represent SE values of data from 80-100 individual seedlings per lines, from 2 independent assays. Different letters indicate significant differences at *P* < 0.05 (Duncan’s test).

To facilitate map-based identification of the *RS24* locus, *rs24* was crossed to wild-type Col-0 plants and a mapping F2 population was produced. Based on the co-segregation of the short period phenotype with genetic markers, the mutation was mapped to the top arm of chromosome 5, between the SSLP (Simple Sequence Length Polymorphism) markers MHF and CIW14. The coding regions of protein coding genes located in this app. 275 kbp region have been amplified by PCR and sequenced. Comparison of sequence data from the wild type and the *rs24* mutant revealed several likely mutagenesis-induced single nucleotide changes, but only one of these was predicted to cause amino acid substitution. This SNP (G-to-A transition at the genomic position 2025743) was located in the seventh exon of the gene encoding *UBP12* and was predicted to cause a serine to phenylalanine substitution at position 327. The mutation lies within the ubiquitin specific protease (USP) domain of the enzyme, and is bordered by the Cys-box and His-box motifs carrying the catalytically essential cysteine and histidine residues, respectively (Fig. 2a). The *UBP12* mRNA accumulated to identical levels in wild-type and *rs24* mutant plants indicating that neither the transcription of *UBP12* nor the turnover of the corresponding mRNA was altered by the mutation (Fig. 2b). For testing the potential effect of the mutation on UBP12 protein accumulation, ectopically expressed epitope-tagged UBP12 proteins were used. The wild-type or the *rs24* mutant version of UBP12 proteins fused to the YFP tag at the C-termini (UBP12^WT^-YFP or UBP12^S327F^-YFP, respectively) were expressed from the 35 S promoter in *ubp12-1* T-DNA insertion null mutant plants carrying the *TOC1:LUC* reporter. A pair of homozygous T3 lines showing very similar levels of wild-type or the mutant *UBP12-YFP* transcripts was selected (Fig. 2c). Western-blots indicated similar UBP12-YFP protein levels in these lines (Fig. 2d) demonstrating that none of the stages of *UBP12* gene expression was affected by the mutation. Quantitative analysis of rhythmic *TOC1:LUC* expression in these lines demonstrated that UBP12^WT^-YFP complemented the short period phenotype of *ubp12-1*, whereas UBP12^S327F^-YFP could not restore wild-type periods (Fig. 2e).

Collectively, these results demonstrate that the circadian clock defect detected in the *rs24* mutant is elicited by the S327F mutation found in the *UBP12* gene and indicate that the mutant phenotype is not caused by the altered level of UBP12, but rather by change in the molecular interactions or function/activity of the enzyme. Accordingly, *rs24* has been designated *ubp12-3*.

### The mutated serine is highly conserved in the orthologues of UBP12

**Fig. 3:**
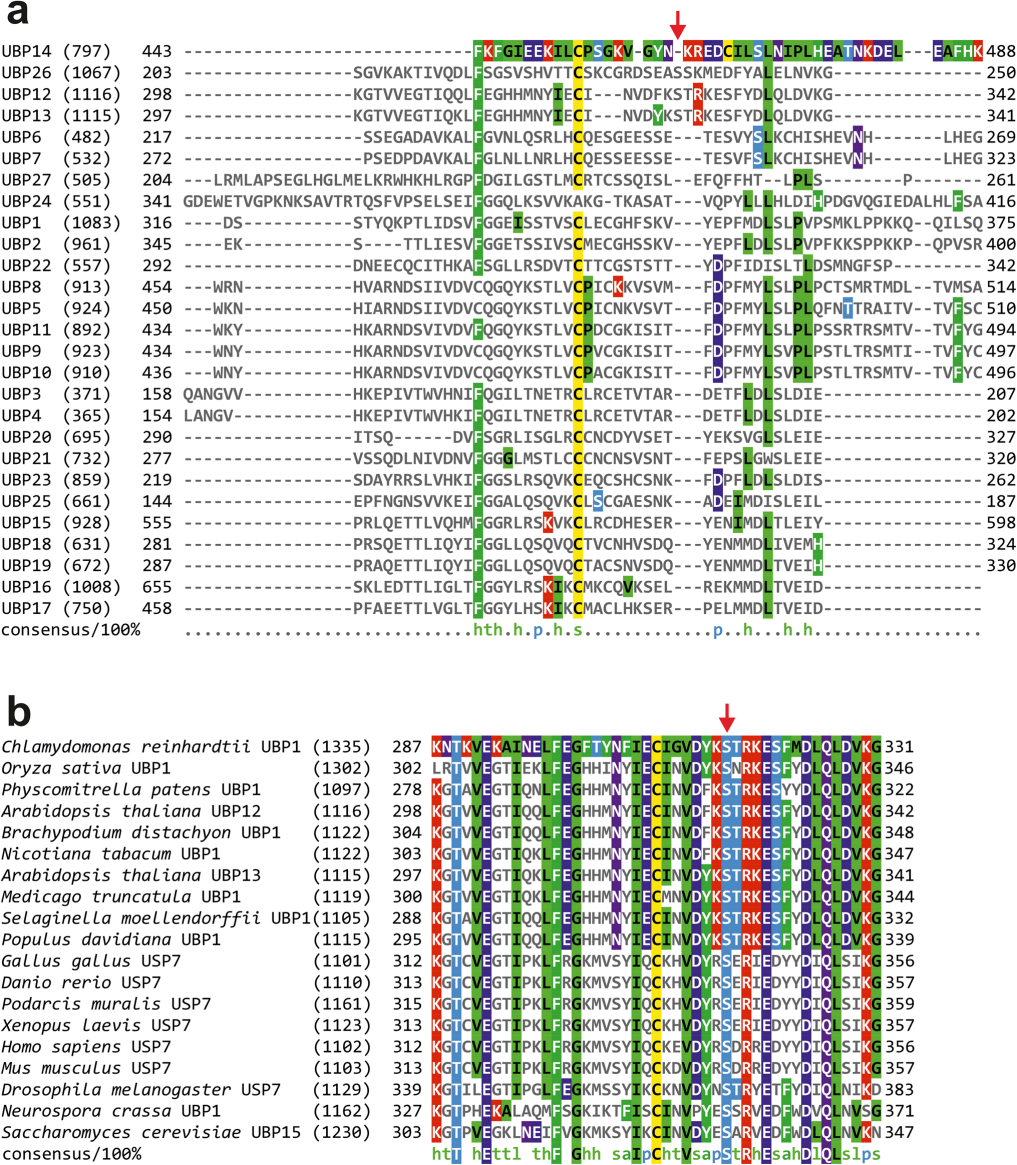
Serine327 of UBP12 is conserved in orthologues. (**a**) Alignments of the USP domains of Arabidopsis UBP protein sequences. The region surrounding S327 in UBP12/13 is shown. S327 in UBP12/13 is indicated by a red arrow. Protein sequences were obtained from TAIR under the following IDs: UBP1: AT2G32780.1, UBP2: AT1G04860.1, UBP3: AT4G39910.1, UBP4: AT2G22310.1, UBP5: AT2G40930.1, UBP6: AT1G51710.1, UBP7: AT3G21280.1, UBP8: AT5G22030.1, UBP9: AT4G10570.1, UBP10: AT4G10590.1, UBP11: AT1G32850.1, UBP12: AT5G06600.1, UBP13: AT3G11910.1, UBP14: AT3G20630.1, UBP15: AT1G17110.1, UBP16: AT4G24560.1, UBP17: AT5G65450.1, UBP18: AT4G31670.1, UBP19: AT2G24640.1, UBP20: AT4G17895.1, UBP21: AT5G46740.1, UBP22: AT5G10790.1, UBP23: AT5G57990.1, UBP24: AT4G30890.1, UBP25: AT3G14400.1, UBP26: AT3G49600.1, UBP27: AT4G39370.1. (**b**) Alignments of sequences of UBP12-like proteins from diverse eukaryotic organisms. The region surrounding S327 in UBP12/13 is shown. S327 in UBP12/13 is indicated by a red arrow. Protein sequences were obtained from UniProt (www.uniprot.org)^39^ under the following IDs: *Chlamydomonas*: A0A2K3DKA3, *Oryza*: Q2R2A3, *Physcomitrella*: A0A7I4AVU6, *Brachypodium*: I1H3I5, *Nicotiana*: A0A1S4D389, *Medicago*: G7I2I7, *Selaginella*: D8S8U6, *Populus*: A0A6M2EY48, *Gallus*: Q6U7I1, *Danio*: I3ISS5, *Podarcis*: A0A670JVL2, *Xenopus*: A0A1L8EQ22, *Homo*: Q93009, *Mus*: Q6A4J8, *Drosophila*: Q9VYQ8, *Neurospora*: A0A0B0ED37, *Saccharomyces*: P50101. All alignments were done by Clustal Omega at the EMBL-EBI server using default parameters (https://www.ebi.ac.uk/jdispatcher/msa/clustalo)^40^. Results were visually optimized by MView at the EMBL-EBI server using default parameters. Numbers in brackets indicate the length of the corresponding full-length protein, whereas numbers at the start and the end of alignments refer to the positions of the first and last amino acid residues shown in the figure.

UBP12 belongs to the subfamily of ubiquitin-specific proteases (UBPs, USPs) consisting of 27 members in Arabidopsis. This subfamily is characterised by the presence of catalytically essential cysteine and histidine residues within the USP domain^23^. UBP13 is the only close homolog of UBP12, in terms of structure and function, as well. UBP12/13 carry a MATH (Meprin And TRAF Homology) domain in the N-terminal part of the proteins, which function as a protein-protein interaction module for binding substrate proteins. None of the other members of the UBP family possess MATH domains^24^. Interestingly, difference between UBP12/13 and other UBPs extends beyond the presence of MATH motifs, since the region of the USP domain containing the mutated serine in UBP12 is also variable. Moreover, the particular serine (S327 in UBP12 and S328 in UBP13) is not present at this position of the other UBPs, maybe with the exception of UBP26 (Fig. 3a).

Using the presence of the MATH domain as a landmark, UBP/USP protein sequences have been collected from a variety eukaryotic organisms ranging from yeast to humans and were aligned. Interestingly, UBP12/13 showed higher similarity to any of these orthologues than to any of Arabidopsis homologs (Fig. 3b). Moreover, the serine residue corresponding to S327 in UBP12 was present in all orthologues listed. The high level conservation of this residue may indicate important regulatory role, which is underlined by our results showing that its mutation results in the alteration of UBP12 function.

### Functional analysis of selected UBP12-targeted signalling pathways indicates increased activity of the enzyme in *ubp12-3*

UBP12 possesses several pleiotropic functions, by affecting ubiquitination level and thus stability or function of master regulatory proteins such as MYC2 or histone H2A acting in different signalling pathways. Testing the output of these pathways can be used as a proxy to investigate the function of UBP12 and its mutant derivative indirectly. This rationale was followed to test if the effect of the mutation is specific to a particular UBP12 target and the associated signalling mechanism, or it has a non-specific general effect and influences the level/function of all/most substrates of UBP12.

**Fig. 4:**
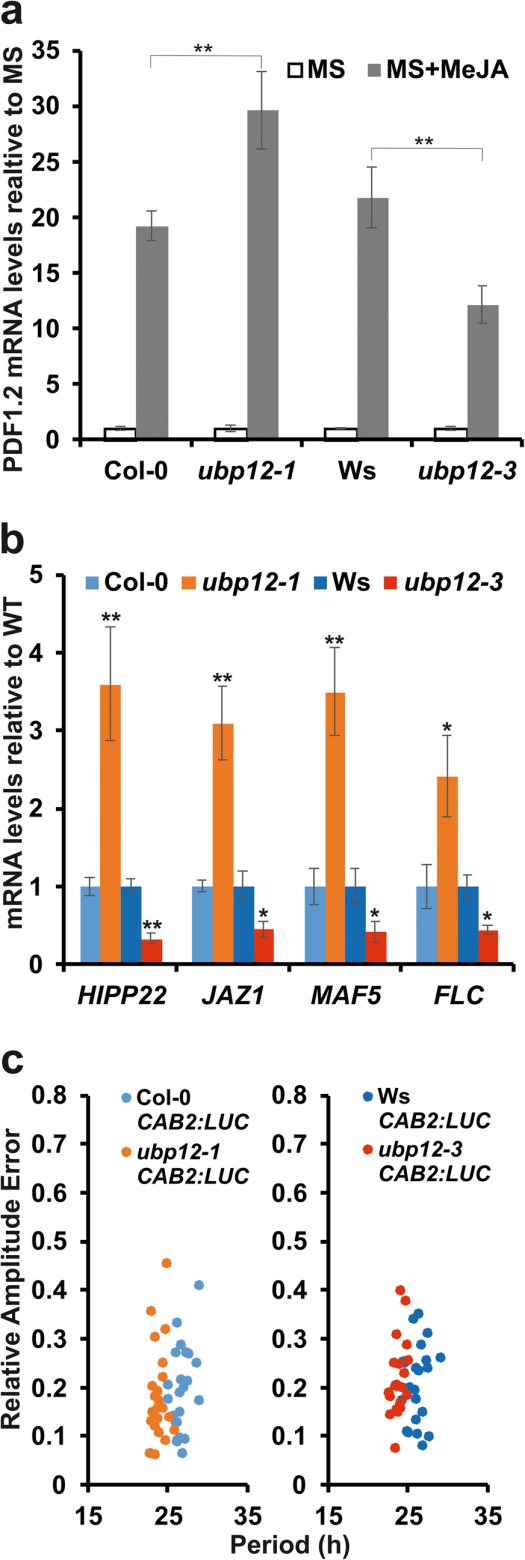
*ubp12-3* resembles the circadian, but not other pleiotropic phenotypes of the T-DNA insertion null mutant *ubp12-1. *(**a**) Col-0, *ubp12-1*, Ws, and *ubp12-3* plants were grown on MS + 1% (w/v) sucrose medium w/o 5 µM methyl jasmonate (MeJA) in 16h light/8h dark photocycles for 14 days and harvested in the middle of the day. Expression of *PDF1.2* gene that was shown to be affected by the ubiquitination level of MYC2 was tested by qPCR reactions. Values normalised to *TUBULIN2/3 *and then to the levels in the corresponding non-induced sample (MS) are shown. Averages of data from three independent experiments are plotted, error bars represent standard error. Asterisks indicate significant differences as determined by Student’s *t* test: ** *P* < 0.01. (**b**) Col-0, *ubp12-1*, Ws, and *ubp12-3* plants were grown on 1/2 MS medium in 16h light/8h dark photocycles for 14 days and harvested in the middle of the day. Expression of selected genes that were shown to be affected by the ubiquitination level of histone H2A (*HIPP22*, *JAZ1*, *MAF5* and *FLC*) was tested by qPCR reactions. Values normalised to *TUBULIN2/3* and then to the levels in the corresponding wild type are shown. Averages of data from three independent experiments are plotted, error bars represent standard error. Asterisks indicate significant differences from the wild type as determined by Student’s *t* test: * *P* < 0.05, ** *P* < 0.01. (**c**) Rhythmic CAB2:LUC luminescence was monitored in Col-0, *ubp12-1*, Ws, and *ubp12-3* plants in continuous red light (10 µmole m^-2^ s^-1^) for 4-5 days. Relative Amplitude Error (RAE) values were plotted against periods. Each dot in the scatter plot represents a single seedling of the given genotype.

UBP12 regulates jasmonate signalling by modulating the level of transcription factor MYC2^19^. MYC2 attenuates the methyl jasmonate (MeJA)-induced expression of *PLANT DEFENSIN 1.2* (*PDF1.2*). Since UBP12 stabilizes MYC2, MeJA-inducion of PDF1.2 is de-repressed in the null mutant *ubp12-1* (Fig. 4a)^19^. In line, we found that MeJa treatment led to stronger *PDF1.2* mRNA induction in the *ubp12-1* null mutants than in the wild-type control. This response to MeJA was also altered in *ubp12-3*, but surprisingly, *PDF1.2* induction was reduced compared to the wild type and especially with the *ubp12-1* mutant (Fig. 4a).

UBP12 regulates chromatin compaction at specific loci via deubiquitinating histone H2A^16^. Removal of mono-ubiquitin moieties from H2A-Ub results in condensation of chromatin and the transcriptional repression of the genes located in the affected regions. Accordingly, several functionally unrelated genes, including *HEAVY METAL ASSOCIATED ISOPRENYLATED PLANT PROTEIN 22* (*HIPP22*), *JASMONATE-ZIM-DOMAIN PROTEIN 1* (*JAZ1*), *MADS AFFECTING FLOWERING 5* (*MAF5*) and *FLOWERING LOCUS C* (*FLC*) were de-repressed in the null mutant *ubp12-1* (Fig. 4b)^16,20^. In contrast, transcription of these genes was clearly repressed in the *ubp12-3* background (Fig. 4b).

Thus, the *ubp12-1* null and *ubp12-3* missense mutants act adversely in the regulation of MYC2- and H2A-dependent genes suggesting that MYC2 and deubiquitinated H2A levels are reduced in the *ubp12-1* and are increased in the *ubp12-3* mutant (relative to the wild type). These observations indicate that UBP12 activity is enhanced in the *ubp12-3* mutant. Apparently contrasting these results, both *ubp12-1* and *ubp12-3* showed a quantitatively similar short period circadian phenotype (Fig. 4c).

**Fig. 5:**
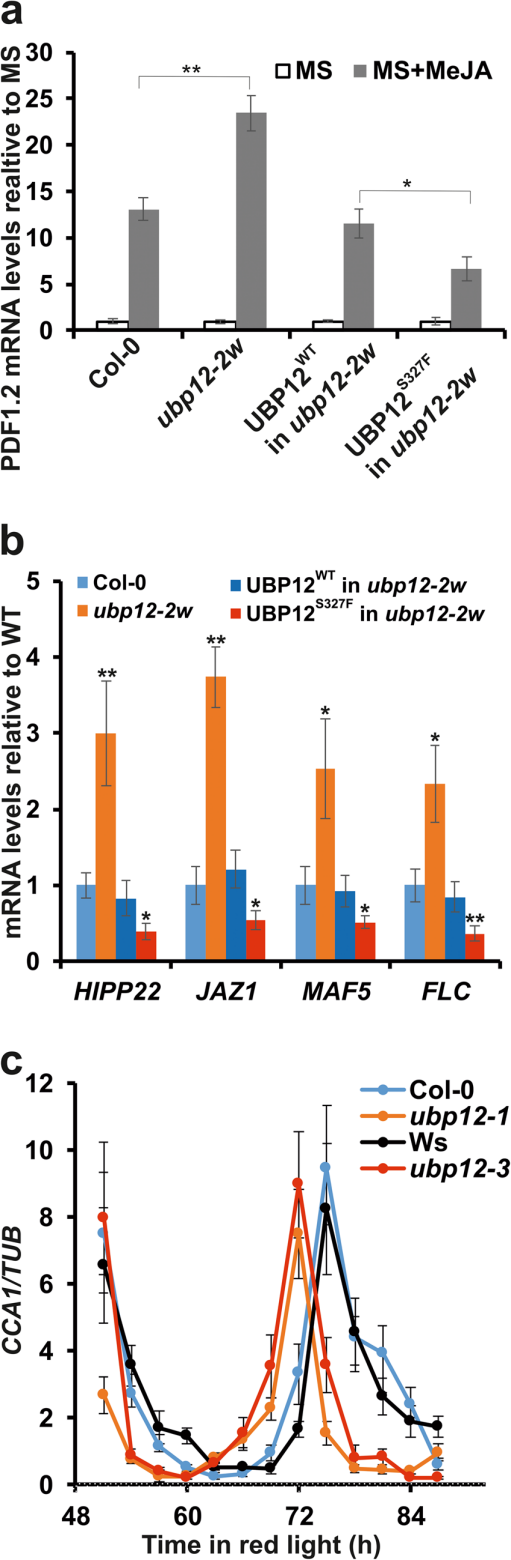
ubp12-3 is a hypermorphic allele of *UBP12.*(**a**) Col-0, *ubp12-2w*, and transgenic*ubp12-2w* plants expressing the UBP12^WT^-YFP or the UBP12^S327F^-YFP were grown on MS + 1% (w/v) sucrose medium w/o 5 µM methyl jasmonate (MeJA) in 16h light/8h dark photocycles for 14 days and harvested in the middle of the day. The two transgenic lines were identical to those used in Fig. 2b-e. Expression of the *PDF1.2* gene was tested by qPCR reactions. Values normalised to *TUBULIN2/3 *and then to the levels in the corresponding non-induced sample (MS) are shown. Averages of data from three independent experiments are plotted, error bars represent standard error. Asterisks indicate significant differences as determined by Student’s *t* test: * *P* < 0.05, ** P < 0.01. (**b**) Col-0, *ubp12-2w*, and transgenic *ubp12-2w* plants expressing the UBP12^WT^-YFP or the UBP12^S327F^-YFP were grown on half-strength MS medium in 16h light/8h dark photocycles for 14 days and harvested in the middle of the day. Expression of *HIPP22*, *JAZ1*, *MAF5* and *FLC* genes was tested by qPCR reactions. Values normalised to *TUBULIN2/3* and then to the levels in the corresponding wild type are shown. Averages of data from three independent experiments are plotted, error bars represent standard error. Asterisks indicate significant differences from the wild type as determined by Student’s *t* test: * *P* < 0.05, ** *P* < 0.01. (**c**) Rhythmic *CCA1* mRNA accumulation was monitored in Col-0, *ubp12-1*, Ws, and *ubp12-3* plants by qPCR assays. Plants were grown in 12 h light / 12 h dark cycles for 7 days and then transferred to continuous red light (10 µmole m^-2^ s^-1^). Samples were harvested at 3-h intervals in the time window of 51-87 h after the transfer to red light. for 4-5 days. Values were normalised to TUBULIN2/3 signals. Averages of data from three independent experiments are plotted, error bars represent standard error.

To further support these findings, expression of the MYC2- and H2A-dependent genes was tested in the transgenic lines expressing the UBP12^WT^-YFP or the UBP12^S327F^-YFP proteins in the *ubp12-2w* mutant. The selected lines expressed the corresponding UBP12 proteins at similar levels, however, UBP12^WT^ complemented the circadian phenotype of *ubp12-2w*, while UBP12^S327F^ apparently did not (Fig. 2d-e). The same lines were used here to test the effect of the ectopically expressed wild type and the mutant UBP12 proteins on the MeJA-induction of *PDF1.2* and the steady-state transcription of *HIPP22*, *JAZ1*, *MAF5* and *FLC* (Fig. 5a-b). UBP12^WT^ complemented the gene expression phenotypes of *ubp12-2w* with transcript levels similar to that of the Col-0 wild type, indicating that this particular line does not overexpress functionally UBP12. In contrast, UBP12^S327F^ expressed at the same level as UBP12^WT^ changed the transcription of the target genes in the direction that indicates functional overexpression of UBP12^19^. These results along with the previous set of experiments demonstrate that UBP12^S327F^ is a hyperactive derivative of UBP12.

The change in the expression of the tested MYC2 or H2A target genes seems to be proportional to the level of UBP12 function. However, the effect of UBP12 on the circadian oscillator appears unique since both the loss and the gain of UBP12 function result in similar short period phenotypes. To reveal the underlying molecular mechanism, we attempted to measure the level of clock proteins that are directly (GI, ZTL) or indirectly (TOC1) affected by UBP12 mediated deubiquitination. However, the use of commercially available antibodies did not result in reproducible and conclusive data. The GI-ZTL complex controls TOC1 protein abundance and *CCA1* is one of the direct targets of the transcriptional repressor TOC1. Thus, transcription level of *CCA1* could be a proxy for the level or activity of the GI-ZTL-TOC1 regulatory module. For this reason, *CCA1* mRNA levels were monitored in *ubp12-1*, *ubp12-3* and the corresponding wild types (Fig. 5c). The short period rhythm of *CCA1* mRNA accumulation was evident in both mutants, but the overall level of *CCA1* transcripts was not different from that of the wild type in any of the mutants. This is in line with earlier results for *ubp12-1*^19^, indicating that regulation of *CCA1* transcription by the GI-ZTL-TOC1 module is not the primary mode of action of UBP12 on the circadian oscillator.

### The circadian defect contributes to the early flowering phenotype of *ubp12* mutants

Our findings described above suggested that for the processes that are directly regulated by UBP12 (e.g. MYC2- and H2A-Ub-dependent processes) *ubp12-1* and *ubp12-3* should show opposite phenotypes, but for the processes that are indirectly regulated by UBP12 (e.g. via the accelerated circadian clock) the two alleles should show at least qualitatively similar phenotypes, since both mutants display similar period shortening compared with wild type.

**Fig. 6:**
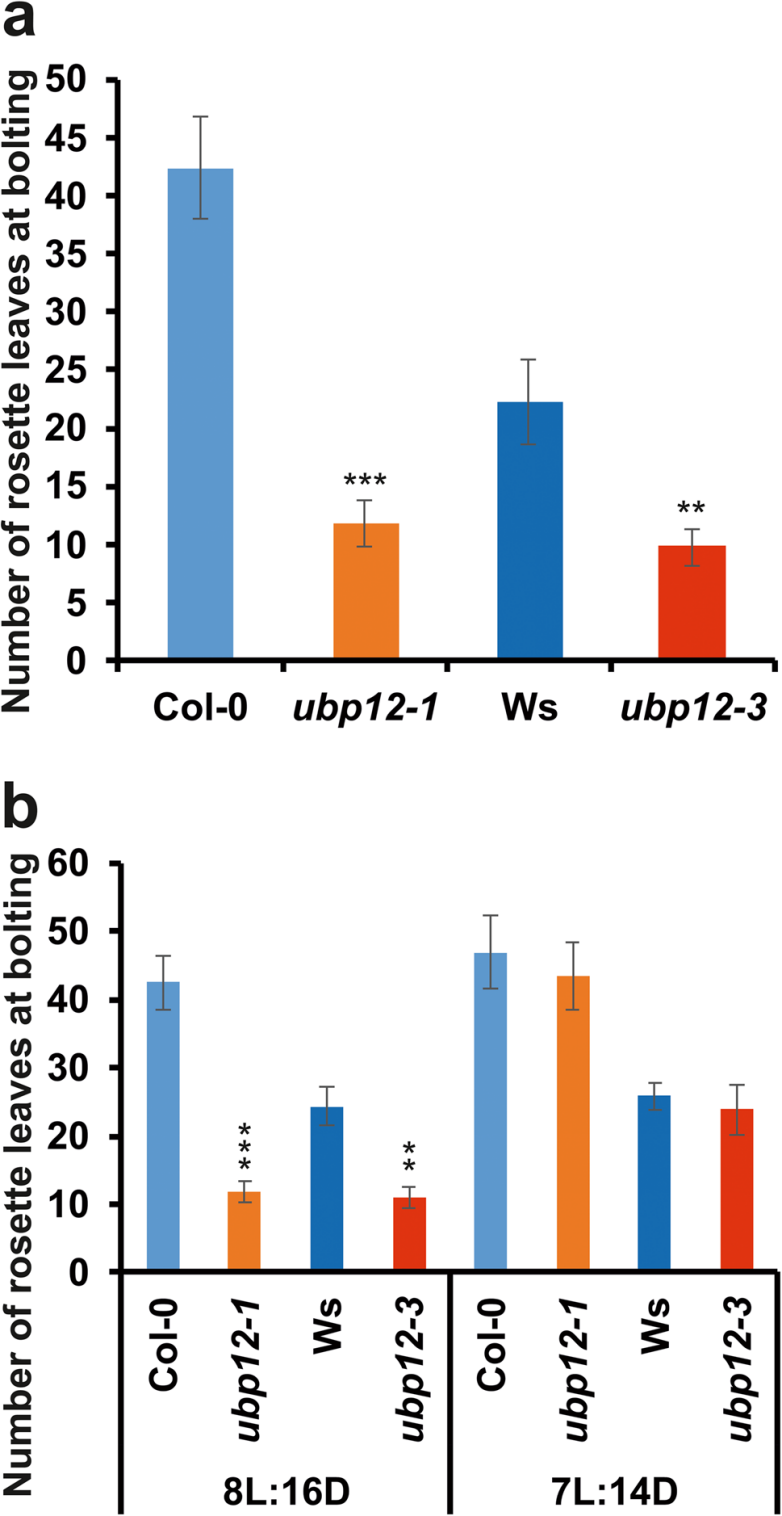
Flowering time phenotype of *ubp12* mutants. Col-0,*ubp12-1*, Ws and *ubp12-3* plants were sowed and grown in soil at 22^o^C in different light/dark regimes. Flowering time was determined as the number of rosette leaves at the time when inflorescence reached the length of about 1 cm. (**a**) Short day conditions with 8h light/16h dark photocycles. (**b**) Testing the effect of T21 cycles on the short day-specific early flowering phenotype of *ubp12* mutants. For this assay plants were grown in 7h light/14h dark photocycles. For comparison, results from the regular (T24) short day conditions are shown along. Graphs represent the averages of three independent experiments, each testing 30-40 individual plants per genotype. Asterisks indicate significant differences from the wild type as determined by Student’s *t* test: * *P* < 0.05, ** *P* < 0.01, *** *P *< 0.001.

One of the best characterised regulatory systems that relies on the timing information provided by the circadian clock is the photoperiodic regulation of flowering. Arabidopsis prefers long days for the initiation of flowering. The actual day length measuring mechanism involves the clock-controlled CONSTANS (CO) transcription factor, which shows the peak of expression at a phase that falls in the light or in the dark under long day (LD) or short day (SD) conditions, respectively. CO protein is stabilized in the light, and thus induces expression of *FLOWERING LOCUS T* (*FT*) in LD conditions. The FT transcription factor acts as a florigen triggering the transformation of the apical meristem into flower meristem^25^. It follows that clock period mutants with altered phasing of circadian rhythms, including the rhythmic expression of CO, often display flowering time phenotypes^26^. In fact, accelerated flowering in *ubp12-1* plants was reported previously, which was explained by the H2A-Ub-dependent chromatin level regulation of key flowering time genes, including *FT*^16^. Our current experiments verified this phenotype of *ubp12-1* and demonstrated that *ubp12-3* displays a very similar early flowering phenotype in SD conditions (Fig. 6a). This indicated that flowering time is probably modulated by UBP12 indirectly, more likely via the function of the circadian clock. To test this, flowering time assays were performed in 7 h light:14 h dark conditions that corresponds to SD conditions with the total day length of 21 h (T21 SD), matching the app. period of *ubp12* mutants. It has been demonstrated that if the period of the day/night cycle is set to match the period of a given circadian mutant with associated flowering phenotype, the correct phase relationship between the environmental cycle and the circadian oscillator is re-established and normal timing of flowering is restored^27^. The results show that the T21 SD cycles eliminated the early flowering phenotype of the *ubp12* mutants (Fig. 6b). This demonstrates that the altered clock function is the primary determinant of the early flowering phenotype of *ubp12* mutants.

### UBP12 affects photomorphogenic responses specifically in red light

**Fig. 7:**
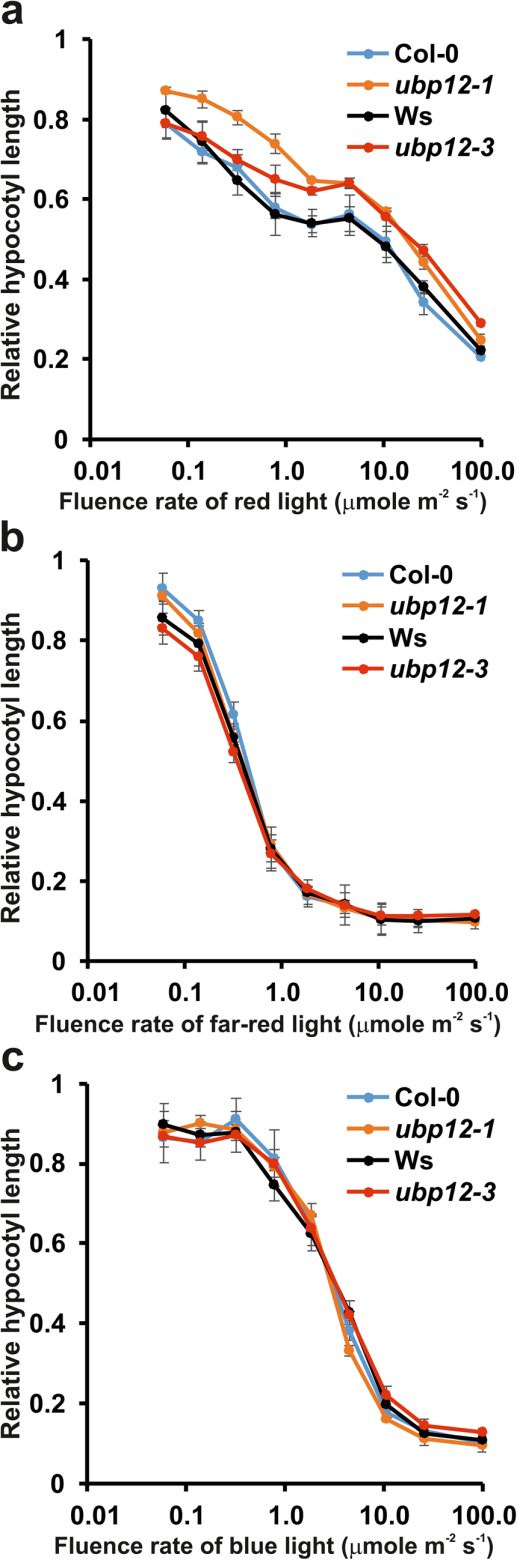
Inhibition of hypocotyl elongation by red light is altered in *ubp12* mutants.Col-0, *ubp12-1*, Ws and *ubp12-3*, seedlings were grown on wet filter paper at different fluences of red (**a**), far-red (**b**) and blue (**c**) light, or in darkness for 4 days. Hypocotyl lengths were measured and normalised to the length of the corresponding dark control. The graphs represent averages of three independent assays. 30-40 seedlings of each genotype were measured in each assays. Error bars represent Standard Error (SE) values.

Photomorphogenic responses represent another group of traits that are usually altered in circadian mutants due the intimate co-action of light- and clock-regulatory pathways^28^. To test this in the *ubp12* mutants, light inhibition of hypocotyl elongation was monitored at different fluence rates of red, far-red and blue light. The hypocotyl elongation response was moderately hyposensitive to red light in the *ubp12* mutants (Fig. 7a). This phenotype was detected over the entire fluence rate in *ubp12-1*, but was not visible at lower fluences in the *ubp12-3* allele. In contrast to red light conditions, the mutants showed no phenotype in far-red (Fig. 7b) or blue (Fig. 7c) light. The similar phenotypes of *ubp12-1* and *ubp12-3* in red light could indicate that the primary cause of hyposensitivity is the altered clock function. However, the complicated nature of this cross-regulation is illustrated by the fact that the short period phenotype is not accompanied by altered hypocotyl elongation in blue light.

### Ser327 has a role in the regulation of the protease activity of UBP12

**Fig. 8:**
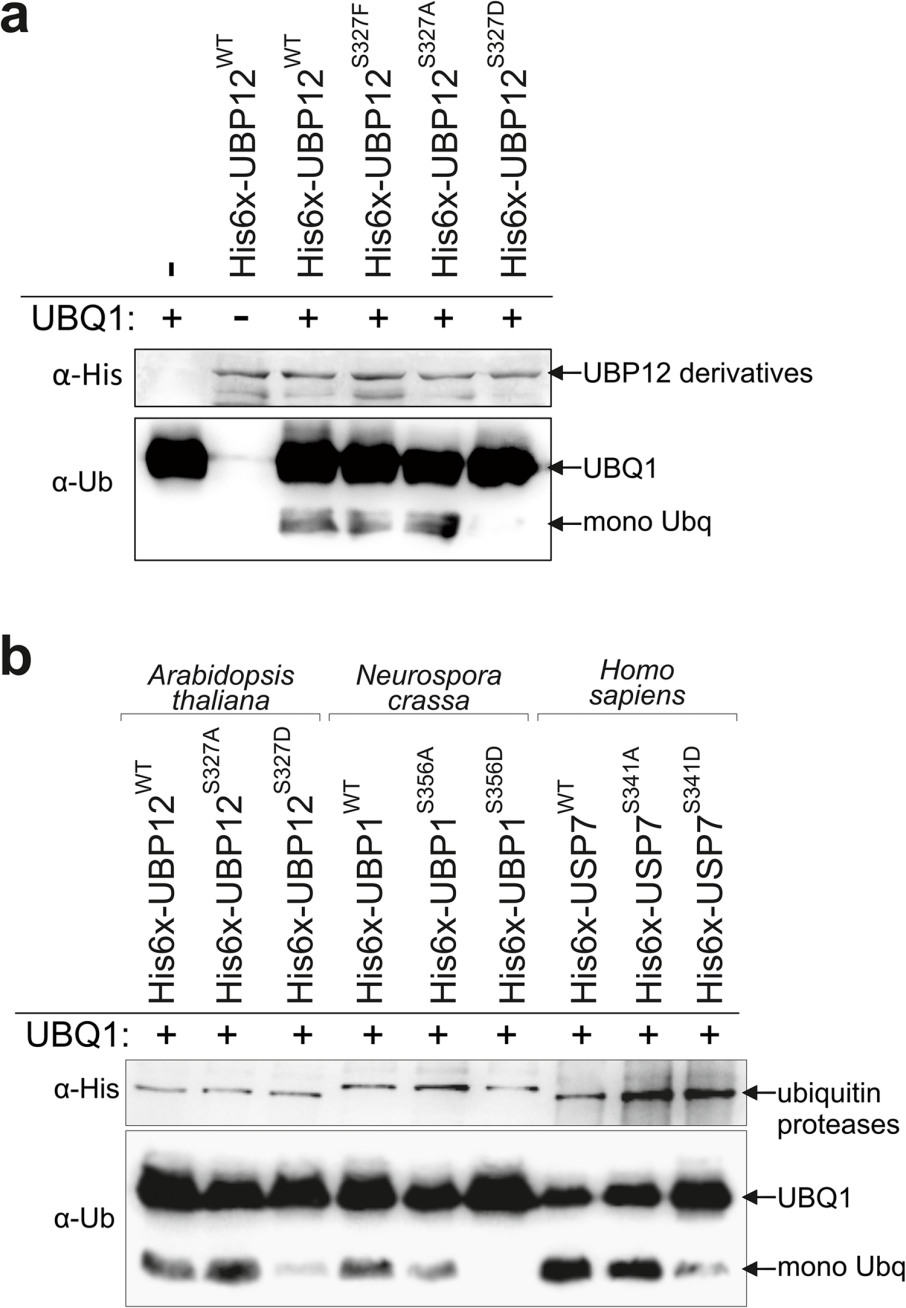
Phosphomimetic mutation of the conserved serine residue inhibits the protease activity of UBP12, NcUBP1 and HsUSP7. (**a**) The UBP12^WT^, UBP12^S327F^, UBP12^S327A^ or UBP12^S327D^ proteins N-terminally fused to the His tag (His6x) were co-expressed with the UBQ1 protein in *E.coli*. Crude extracts of bacterial cultures were loaded on SDS-PAGE gels. Cleavage of single ubiquitin peptides (mono Ub) off the UBQ1 extension protein was detected by anti-ubiquitin antibodies (α-Ub) on Western blots. Levels the expressed UBP12 derivatives was tested by anti-His6x antibodies (α-His). (**b**) The *Arabidopsis thaliana* UBP12^WT^, UBP12^S327A^ or UBP12^S327D,^the*Neurospora crassa* UBP1^WT^, UBP1^S356A^ or UBP1^S356D^, and the human USP7^WT^, USP7^S341A^ or USP7^S341D^ proteins fused to the His tag (His6x) were co-expressed with the UBQ1 protein in *E. coli*. Ubiquitin protease activity was tested and detected as in (**a**). Uncropped blots are shown in Supplementary Information 1b. The assays were repeated at least 3 times and representative blots are shown.

Our data indicated that the S327F mutation affected the function of UBP12 towards several different targets suggesting that a general, core feature of the enzyme, most probably the ubiquitin protease activity, was enhanced. In order to test this directly, UBP12^WT^ and UBP12^S327F^ were co-expressed with the Arabidopsis ubiquitin extension protein UBQ1 in bacterial cells. UBQ1 is a natural substrate of ubiquitin proteases and consists of a single ubiquitin peptide at the N-terminal part of the protein fused to a smaller C-terminal domain of unknown function via an α-peptide bond. Several ubiquitin-specific proteases, including UBP12, were shown to cleave UBQ1 between the N-terminal and C-terminal domains releasing a single ubiquitin peptide, which can be readily detected by Western blots^15,24^. This activity of the wild-type UBP12 has been verified (Fig. 8a) by our assay, which showed similar protease activity for UBP12^S327F^ as well. This indicates that the identified mutation per se does not increase the enzymatic activity of UBP12.

Serine residues are one of the most frequently phosphorylated amino acids in eukaryotes. It has been long established that the effect of un/phosphorylation of a given residue on the biochemical/functional properties of the protein can be mimicked by particular amino acid replacements. For example, alanine (A) or phenylalanine (F) mimic non-phosphorylated serine, whereas glutamic acid (E) or aspartic acid (D) structurally resembles phosphorylated serine. We reasoned that in case S327 is phosphorylated and it affects the activity of the enzyme, the S327F mutation could prevent this modification and thus modulate UBP12 function *in planta*.

To investigate this hypothesised function of S327, two additional derivatives, UBP12^S327A^ and UBP12^S327D^ have been expressed and tested in the bacterial system. UBP12^S327A^ showed protease activity similar to those of UBP12^WT^ and UBP12^S327F^. However, the cleavage efficiency of UBP12^S327D^ was significantly lower compared with the other derivatives (Fig. 8a).

Taken together, these data suggest that phosphorylation at S327 inhibits the activity of UBP12. Therefore, we propose that the S327F mutation could cause apparent over-functioning of UBP12 *in planta* by preventing inhibitory phosphorylation.

The remarkable conservation of this particular serine residue (S327 in UBP12) among UBP12 orthologues prompted us to test the effect of its directed mutation on the enzymatic activity of ubiquitin proteases in other organisms. To this end we obtained the cDNA of UBP1 from *Neurospora crassa* (NcUBP1) and the human USP7 (HsUSP7) and generated the S/A and S/D mutations at the conserved serine residues that were S356 in NcUBP1 and S341 in HsUSP7 (Fig. 3a). The wild-type and phosphomutant variants were then tested for their efficiency in cleaving Ubq from UBQ1. Figure 8B demonstrates that specifically the S/D replacement reduced the protease activity of both NcUBP1 and HsUSP7. This observation suggests that phosphorylation of the conserved serine residue could be a common mechanism controlling the function of UB12-like ubiquitin proteases.

## Discussion

Ubiquitination is a reversible posttranslational modification, during which ubiquitin ligase complexes catalyse the conjugation of ubiquitin peptide(s) to target proteins, whereas ubiquitin proteases can counterbalance their function by removing ubiquitin tags from targets. Monoubiquitination usually alters the activity or subcellular localization of the modified proteins or affects their ability to interact with other proteins. On the other hand, polyubiqitination usually marks proteins for degradation via the 26 S proteasome, and represents a finely tuned mechanism for setting the level of the components of various signalling systems.

We have isolated a novel missense allele (*ubp12-3*) of the ubiquitin protease UBP12 involved in the regulation of the circadian clock, jasmonate signalling and epigenetic control of gene expression as well^17^. Comparative characterization of *ubp12-3* along with the *ubp12-1* null allele revealed apparently contrasting results. Regarding circadian period and circadian clock-related functions (e.g. flowering time determination) the two alleles showed similar phenotypes, but they exerted opposite effects on MYC2-mediated jasmonate induced gene expression or H2A-dependent epigenetic control of transcription. Our results suggest that UBP12 activity is enhanced in *ubp12-3* due to the prevention of an inhibitory phosphorylation, explaining the opposite effects of *ubp12-1* and *ubp12-3* on MYC2- and H2A-releated gene expression. We propose that the similar effects of the null and the missense alleles on clock-related processes originate from the functional properties of the cognate circadian target of UBP12.

We have identified a novel missense allele of UBP12 (designated *ubp12-3*) based on its short period circadian phenotype. Since the mutation (changing serine to phenylalanine at amino acid position 327, S327F) is located in the USP domain of the protein and it caused a clock phenotype very similar to that of the null mutant allele, it was assumed to abolish the protease activity of the enzyme. To verify the general loss (or reduction) of the enzymatic activity of UBP12^S327F^, expression of marker genes, indirectly reporting the ubiquitination state of two additional and unrelated UBP12 targets, MYC2 and histone 2 A, were tested in the *ubp12-1* null and the currently identified *ubp12-3* mutants. The transcript levels of these genes were clearly altered in both *ubp12-1* and *ubp12-3* supporting the idea that the S327F mutation amends the generic function of UBP12. However, we detected the opposite direction of the change of gene expression in the null versus the missense alleles, indicating that UBP12^S327F^ is not a hypo-, but rather a hypermorphic derivative of the enzyme.

The over-functioning nature of the *ubp12-3* allele is also consistent with the observed photoperiodic flowering phenotype. We showed that both *ubp12-1* and *ubp12-3* flower earlier than the wild type plants in short day conditions. If the flowering phenotype had been caused directly by changes in UBP12 activity, the two alleles would have shown opposite shifts in timing of flowering. This leads to the conclusion that early flowering probably arose from the short period clock phenotype. Indeed, the early flowering phenotypes could be rescued by fitting the period of the environmental light/dark cycle to the period of the clock in the mutants, providing another line of evidence that these traits were caused primarily by the accelerated clock function. On the other hand, the considerations above could lead to the conclusion that all phenotypes that are common in *ubp12-1* and *ubp12-3* are probably the result of the clock defect (i.e. short periods) and not the altered activity of UBP12. Hence, the similar hyposensitive photomorphogenic phenotype of the mutants could also be the consequence of the similar short period clock phenotypes.

How could both the decrease (*ubp12-1*) and the increase (*ubp12-3*) of UBP12 activity result in very similar circadian phenotypes?

GI is an important clock protein that rhythmically affect the stability and accumulation of other clock components like ZTL and TOC1, and thus regulates period of the clock^12,14^. The stability and therefore level of GI is influenced by ubiquitination, which is directly affected by UBP12^14^. Interestingly, it has been reported that *gi* null mutants and GI over-expressing plants could display similar circadian phenotypes in terms of periods shorter than that of the wild type plants^29^, indicating that alterations from the optimal GI levels in either directions result in similar period phenotypes. This could explain how hyper- or hypo-ubiquitination possibly resulting in reduced or increased GI protein levels in *ubp12-1* or *ubp12-3*, respectively, result in similar short period phenotypes.

Alternatively, this phenomenon could be explained if the S327F mutation specifically and differentially modulates the interaction of UBP12 with its different targets: weakens the binding of UBP12 to the circadian target GI, but strengthens its interaction with MYC2 and histone H2A. However, the mutation is located outside of the MATH domain, which is essential and sufficient to make physical interaction with target proteins, thus making this explanation unlikely. To provide experimental support for these hypotheses we aimed to test ubiquitination state/abundance of GI and ZTL, but this was prevented by technical limitations.

To test directly the effect of the S327F mutation on the enzymatic activity of UBP12, UBP12^WT^ and UBP12^S327F^ proteins were co-expressed with the ubiquitin extension protein UBQ1 in bacteria and the cleavage of the single ubiquitin unit off the UBQ1 was monitored by Western blots. However, this biochemical assay showed no differences in the protease activities of the wild-type and the mutant UBP12 proteins, which is contrast to the apparently increased activity of UBP12^S327F^ observed *in planta*. This indicated that the mutation may cause a change in the structure or the function of UBP12 that could be manifested in plant cells, but not in bacteria. Such a change could be, for example, a specific phosphorylation, which frequently occurs on serine residues in eukaryotes. In fact, the analysis of protease activity of non-phosphorylatable (S327A) and phosphomimetic (S327D) mutant variants of UBP12 suggested that phosphorylation on S327 may inhibit the biochemical function of the enzyme. The S327A mutation, similar to the originally identified S327F, had no apparent effect on enzymatic activity, which is consistent with the presumed lack of S327 phosphorylation in bacteria.

The residue S327 of UBP12 and its sequence context is also present in the close homolog UBP13 (S326) (identity: 91%), but is missing from the all the other Arabidopsis UBP proteins, which also lack the MATH domain. Intriguingly, this serine residue is conserved in the UBP12 orthologues, which possess the MATH domain, indicating that the regulation of enzymatic activity of MATH domain-containing UBP12-like proteins by phosphorylation could be phylogenetically conserved. In fact, the ubiquitin protease domain of UBP12 shows significantly higher identity to that of the human orthologue HsUSP7 than to any of the Arabidopsis homologs. The crystal structure of HsUSP7 suggested that this particular serine residue (S341 in HsUSP7) plays a role in fixing the ubiquitin moiety of substrate proteins by hydrogen-bonds^10^. Therefore, it is possible that phosphorylation at this position interferes with ubiquitin binding and thus reduces the protease activity of the enzyme. Our data showing that the S341D mutation, but not the S341A replacement reduced the enzymatic activity of HsUSP7 are in line with this hypothesis. Similar to UBP12/13, HsUSP7 has a highly pleiotropic function acting on a number of substrates. HsUSP7 is involved in the initiation of cancer and tumorigenesis by affecting stability of p53^30^. Interestingly, the mouse equivalent of HsUSP7 was shown to modulate the period of the mammalian circadian clock via regulating the stability of the cryptochrome 1 and 2 clock proteins^31^. The knock-out of USP7 in mice is lethal^32^ underlining the importance of the function of this protease. On the other hand, various deletion or substitution mutant alleles of HsUSP7 have been associated with a complex neurodegenerative disease, the Hao-Fountain syndrome^33^. Even in the case of this intensively studied mammalian UBP12 orthologue very limited information is available on the regulatory role of phosphorylation. HsUSP7 is phosphorylated on serine residues at positions 18 and 963^34^. S18 is located in the MATH domain of the protein and its phosphorylation inhibits the binding to p53^35^, whereas the functional relevance of S963 phosphorylation remains unknown. Since we show that targeted manipulation of the conserved serine residue in NcUBP1 and HsUSP7 results in the same change in protease activity as observed in AtUBP12, our work represents the first report predicting a role for phosphorylation in regulating the enzymatic activity of MATH domain-containing ubiquitin-specific proteases, even in distantly related species.

We propose the following hypothesis to explain the effect of the mutation identified in *ubp12-3* and to suggest a mechanism for the regulation of protease activity by phosphorylation. In wild-type plant cells, a certain portion of UBP12^WT^ is probably phosphorylated on S327 and therefore inactivated, because fixing the ubiquitin in the binding pocket is impaired. In *ubp12-3* UBP12^S327F^ mutant proteins are expressed at a level comparable to that of UBP12^WT^ in the wild-type plants, and the protease activity of UBP12^S327F^ does not differ significantly from that of the unphosphorylated UBP12WT. However, since phosphorylation is blocked at position 327 in the mutant, the entire UBP12 population is enzymatically active that results in a higher overall UBP12-specific activity in the mutant.

## Methods

### Plant materials, growth conditions and light treatments

*Arabidopsis thaliana* plants of the Columbia-0 (Col-0) and the Wassilevskija-2 (Ws-2) accessions were used in this study. The Ws-2 line harbouring the *CAB2:LUC* marker that was used for EMS-induced mutagenesis resulting in the *red screen 24* (*rs24*) mutant and the procedures for screening have been described^21,22^. The *ubp12-2w* and *ubp12-1* alleles are T-DNA insertion alleles of *UBP12* and were isolated from the GABI-Kat collection (line ids: GK-742C10 and GK-244E11, respectively)^15^. The *TOC1:LUC* reporter construct and the selected transgenic Col-0 line harbouring this marker have been described^36^. The TOC1:LUC marker was introgressed in *ubp12-1* by crossing.

To produce the *35 S: UBP12*^*WT*^*-YFP* gene construct, *UBP12* cDNA molecules without the translational termination codons were PCR-amplified from a size-selected cDNA library (CD4-16, TAIR) and cloned as *Xba*I - *Ehe*I fragments between the 35 S promoter of the Cauliflower Mosaic Virus and the Yellow Fluorescent Protein (YFP) gene in the modified pPCV812 binary vector^36^ at *Xba*I - *Sma*I sites. The mutation for Ser327Phe substitution (S327F) was introduced by using the QuickChange Lightning Site-Directed Mutagenesis Kit (Agilent, # 210518) according to the manufacturer’s instructions. The mutant version of the gene was used to produce the *35 S: UBP12*^*S327F*^*-YFP* gene construct.

In general, surface sterilized seeds were sowed on solidified Murashige and Skoog (MS) media supplemented with 3% (w/v) sucrose. Seedlings were grown in 12 h white light / 12 h dark conditions at 22^o^C for 7 days before starting specific assays or harvesting them. White light during growth/entrainment was provided by LUMILUX XT T8 L 36 W/865 (Osram) fluorescent tubes at 70–100 µmol m^−2^ s^−1^ fluence rate. Red (λmax = 660 nm), far-red (λmax = 735 nm) and blue light (λmax = 470 nm) were provided by SNAP-LITE LED light sources (Quantum Devices, WI, USA).

To analyse MYC2-dependent jasmonate-induced expression of PDF1.2, plants were grown on MS media with 1% (w/v) sucrose (control) or MS media with 1% (w/v) sucrose supplemented with 5 µM methyl-jasmonate in 16 h white light / 8 h dark conditions at 22^o^C for 14 days.

To analyse histone 2 A-dependent gene expression, plants were grown on half-strength MS media without sucrose in 16 h white light / 8 h dark conditions at 22^o^C for 14 days.

### Analysis of gene expression

Total RNA was isolated with the NucleoSpin RNA Plant and Fungi Mini Kit (Macherey-Nagel, # 740120.250). 1 µg total RNA was used as template for reverse transcription done with the RevertAid First Strand cDNA Synthesis Kit (Thermo Scientific, # K1622). cDNA samples were diluted 1:5 and used as templates in quantitative real-time PCR assays employing qPCRBIO SyGreen Mix Hi-ROX mastermix (PCR Biosystems Ltd.) and an ABI Prism 7300 Real Time PCR System (Life Technologies). All procedures were performed according to the manufacturer’s instructions. The standard curve method was used for calculation of relative expression levels. Sequences of all qPCR primers are provided in Supplemental Table 1.

Total protein extraction, Western blot analysis and detection of YFP fusion proteins were done essentially as described^37^ except that a horse radish peroxidase-conjugated secondary anti-mouse antibody (Thermo Scientific) was used. Chemiluminescent signals were detected and quantified as described^38^. The assays were repeated two or three times and representative data are shown.

### Ubiquitin protease assays in *E. coli*

The S327A and S327D derivatives of the UBP12 gene were generated by QuickChange Lightning Site-Directed Mutagenesis Kit (Agilent, # 210518) in the same manner as for S327F above. The four UBP12 variants (*UBP12*^*WT*^, *UBP12*^*S327F*^, *UBP12*^*S327A*^, *UBP12*^*S327D*^) have been re-amplified in order to add 5’ *Sac*I and 3’ *Not*I sites and were cloned in pET32a vectors accordingly. These variants were co-expressed with the substrate UBQ1 in *E. coli* BL21 cells as described^24^. Crude bacterial lysates were loaded on 15% SDS polyacrylamide gels, transferred to PVDF membrane. UBQ1 and the cleaved-off single Ubq proteins were detected by an anti-Ubq antibody (P4D1, Santa Cruz). Alternatively, the crude extracts were loaded on 7% SDS PA gels to test the levels of the expressed UBP12 variants utilizing the N-terminal His(6x)-tag from pET32a.

The coding region of NcUBP1 was amplified on a cDNA library prepared from total RNA isolated from *Neurospora crassa* 74-OR23-1VA (FGSC 2489). Primers were designed to aid cloning in pET32 vectors via *BamH*I (5’) and *Pst*I (3’) sites.

The HsUSP7 ORF cloned in pGEM-T vector was purchased from Sino Biological (cat.#: 1 S-HG11681-G) and was amplified with *BamH*I (5’) and *Hind*III (3’) sites to facilitate cloning in pET32A. The phosphomutant variants of NcUBP1 (NcUBP1^S356A^, NcUBP1^S356D^) and HsUSP7 (HsUSP7^S341A^, HsUSP7^S341D^) were created as those of UBP12 above. All NcUBP1 and HsUSP7 derivatives were expressed and processed as those of UBP12 above. Oligonucleotides used for cloning and mutagenesis are listed in Supplemental Table 1.

### Luminescence assays

Luciferase activity was measured by measuring single seedlings with an automated luminometer (TopCount NXT, Perkin Elmer) for 7 days as described previously^22^. All rhythm data were analyzed with the Biological Rhythms Analysis Software System 2 (BRASS2, had been available at http://www.amillar.org, replaced by BioDare2, available at https://biodare2.ed.ac.uk/), running fast Fourier transform nonlinear least-squares estimation. Variance-weighted mean periods within the circadian range (15–40 h) and SEMs were estimated as described, from 12 to 36 traces per genotype. Experiments were repeated three or four times.

### Measurement of flowering time

Seeds were sown on soil and grown in T24 short day (8 h white light / 16 h dark) or T21 short day (14 h white light / 7 h dark) conditions at 22^o^C. Flowering time was recorded as the number of rosette leaves at the time when inflorescences reached 1 cm height. Experiments were repeated twice or three times using 30 to 40 plants per genotype.

## Supplementary Information

Supplementary Material 1.

Supplementary Material 2.

## Data Availability

The datasets generated and/or analysed during the current study are available in the BioDare2 repository, https://biodare2.ed.ac.uk/experiments, ID: 28943.
