# Author Correction: Forward genetic approach identifies a phylogenetically conserved serine residue critical for the catalytic activity of UBIQUITIN-SPECIFIC PROTEASE 12 in Arabidopsis

**DOI:** 10.1038/s41598-024-79318-x

**Published:** 2024-11-20

**Authors:** Anita Hajdu, Dóra Vivien Nyári, Éva Ádám, Yeon Jeong Kim, David E. Somers, Dániel Silhavy, Ferenc Nagy, László Kozma-Bognár

**Affiliations:** 1 Department of Genetics, Faculty of Sciences and Informatics, University of Szeged, Szeged, 6726 Hungary; 2 Institute of Plant Biology, Biological Research Centre, Hungarian Research Network (HUN-REN), Szeged, 6726 Hungary; 3 Department of Medical Genetics, Faculty of Medicine, University of Szeged, Szeged, 6720 Hungary; 4 Doctoral School in Biology, Faculty of Science and Informatics, University of Szeged, Szeged, 6726 Hungary; 5 Department of Molecular Genetics, Ohio State University, Columbus, OH USA

Correction to: *Scientific Reports* 10.1038/s41598-024-77232-w, published online 25 October 2024

The original version of this Article contained an error in the Acknowledgements section.

“We thank Dr. Judy Callis (University of California, Davis) for kindly sharing plasmids pACYC184 and p8185. This study was supported by grants from the National Research, Development and Innovation Office (Hungary). Grant numbers: AN-128740 and K-134567 to L.K-B., PD-138963 to A.H., and K-139349 to D.S. A.H. was supported by a fellowship programme (KGYNK) from the Hungarian Academy of Sciences.”

now reads:

“We thank Dr. Judy Callis (University of California, Davis) for kindly sharing plasmids pACYC184 and p8185. This study was supported by grants from the National Research, Development and Innovation Office (Hungary). Grant numbers: AN-128740 and K-134567 to L.K-B., PD-138963 to A.H., and K-139349 to D.S. A.H. was supported by a fellowship programme (KGYNK) from the Hungarian Academy of Sciences. Y.J.K. and D.E.S. were supported by the National Institutes of Health (United States), grant number: R35GM136400.”

The original Article has been corrected.

