# Supplementary material for: Forward genetic approach identifies a phylogenetically conserved serine residue critical for the catalytic activity of UBIQUITIN-SPECIFIC PROTEASE 12 in Arabidopsis
Source: Sci Rep. 2024 Oct 25;14:25273. doi: 10.1038/s41598-024-77232-w (PMC11511944; doi:10.1038/s41598-024-77232-w)
Supplement: Supplementary file 1 — Supplementary Material 1. [file 41598_2024_77232_MOESM1_ESM.pdf]

Table S1. Oligonucleotides used in this work	
**Primers for qPCR assays**	
**Primer ID**	**Sequence (5' to 3')**
**UBP12 RT-1 F**	AGTTCGCAGTTTCCGTATCC
**UBP12 RT-1 R**	TTGTCTCTTTGCCCAAATCCA
**HIPP22 RT-1 F**	GGAAGAAACGTAAAGTCATGCAG
**HIPP22 RT-1 R**	GTGCATCTTTCTGTTCACTTCC
**JAZ1 RT-1 F**	TAGCCGGAGATTTACTGGGA
**JAZ1 RT-1 R**	GAGTTGCCTAAAGTTCCATTGAC
**MAF5 RT-1 F**	GCAAACTCTACAACTCCTCCT
**MAF5 RT-1 R**	TTTGTCTTCAAGATCCTGCCA
**FLC-K1 RT F**	TGAAAGAAGAGAACCAGGTTTTGG
**FLC-K1 RT R**	GTCCAGCAGGTGACATCTCCA
**YFP RT Fwd**	CACAAGCTGGAGTACAACTA
**YFP RT Rev**	GATCTTGAAGTTCACCTTGATG
**TUB 2/3 RT Fwd**	CCAGCTTTGGTGATTTGAAC
**TUB 2/3 RT Rev**	CAAGCTTTCGGAGGTCAGAG
**Oligonucleotides for cloning and mutagenesis**	
**Oligonucleotid ID**	**Sequence (5' to 3')**
**UBP12_CDS_Fwd_XbaI_pPCV812**	ATATCTAGAATGACTATGATGACTCCGCCTC
**UBP12_CDS_Rev_S-_EheI_pPCV812**	ATAGGCGCCATTGTATATTTTTACCGGCTTCTCG
**UBP12_CDS_F_SacI_pET32a**	ATAGAGCTCATGACTATGATGACTCCGCCTC
**UBP12_CDS_R_S+_NotI_pET32a**	ATAGCGGCCGCCTAATTGTATATTTTTACCGGCTTCTC
**UBP12_S327F_Fwd**	GTGCATAAATGTAGATTTTAAATTTACACGGAAAGAATCATTTTACGACC
**UBP12_S327F_Rev**	GGTCGTAAAATGATTCTTTCCGTGTAAATTTAAAATCTACATTTATGCAC
**UBP12_S327A_Fwd**	GTGCATAAATGTAGATTTTAAAGCTACACGGAAAGAATCATTTTACGACC
**UBP12_S327A_Rev**	GGTCGTAAAATGATTCTTTCCGTGTAGCTTTAAAATCTACATTTATGCAC
**UBP12_S327D_Fwd**	GCATAAATGTAGATTTTAAAGATACACGGAAAGAATCATTTTACGACCTTCAGC
**UBP12_S327D_Rev**	GCTGAAGGTCGTAAAATGATTCTTTCCGTGTATCTTTAAAATCTACATTTATGC
**NcUBP1_F_BamHI_pET32a**	ATAGGATCCATGGATGACCAAAACCCCC
**NcUBP1_R_PstI_pET32a**	ATACTGCAGTTATCCCTTGAGGAACAAGTCACC
**NcUBP1_S356A_F**	CAACGTTCCGTACGAAGCCAGTCGAGTTGAAGATTTC
**NcUBP1_S356A_R**	GAAATCTTCAACTCGACTGGCTTCGTACGGAACGTTG
**NcUBP1_S356D_F**	CAACGTTCCGTACGAAGACAGTCGAGTTGAAGATTTC
**NcUBP1_S356D_R**	GAAATCTTCAACTCGACTGTCTTCGTACGGAACGTTG
**HsUSP7_F_BamHI_pET32a**	ATAGGATCCATGAACCACCAGCAGCAG
**HsUSP7_R_HindIII_pET32a**	ATAAAGCTTTCAGTTATGGATTTTAATGGCCT
**HsUSP7_S341A_F**	GAAGTAGACTATCGGGCTGATAGAAGAGAAGATTATTATG
**HsUSP7_S341A_R**	CATAATAATCTTCTCTTCTATCAGCCCGATAGTCTACTTC
**HsUSP7_S341D_F**	GAAGTAGACTATCGGGATGATAGAAGAGAAGATTATTATG
**HsUSP7_S341D_R**	CATAATAATCTTCTCTTCTATCATCCCGATAGTCTACTTC

Supplemental Table 1. Primers and oligonucleotides used in qPCR assays, or for cloning and mutagenizing *UBP12*, *NcUBP1* and *HsUSP7*.
