# Supplementary material for: Forward genetic approach identifies a phylogenetically conserved serine residue critical for the catalytic activity of UBIQUITIN-SPECIFIC PROTEASE 12 in Arabidopsis
Source: Sci Rep. 2024 Oct 25;14:25273. doi: 10.1038/s41598-024-77232-w (PMC11511944; doi:10.1038/s41598-024-77232-w)
Supplement: Supplementary file 2 — Supplementary Material 2. [file 41598_2024_77232_MOESM2_ESM.pdf]

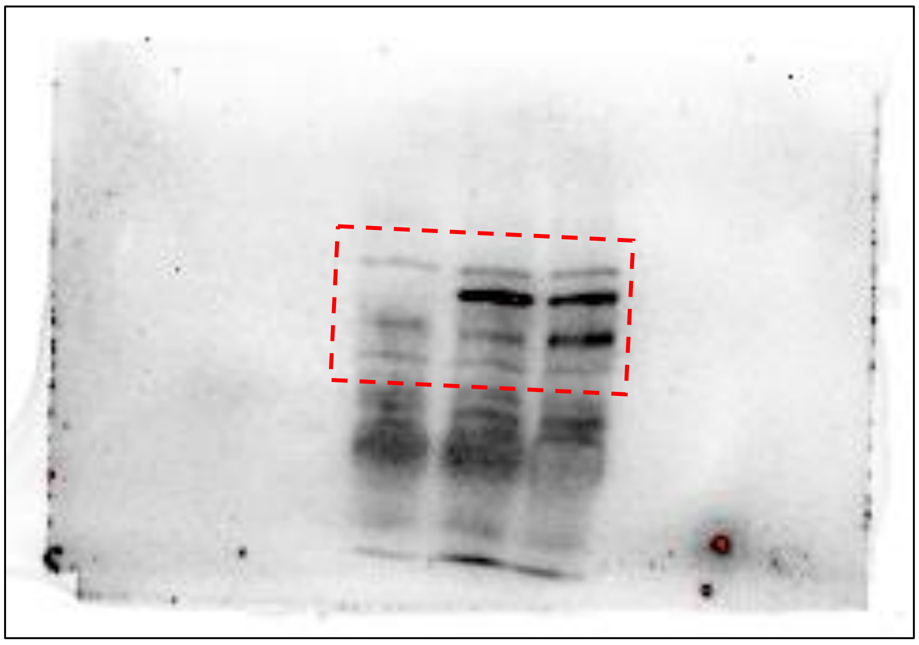

Figure 2d, α-GFP blot

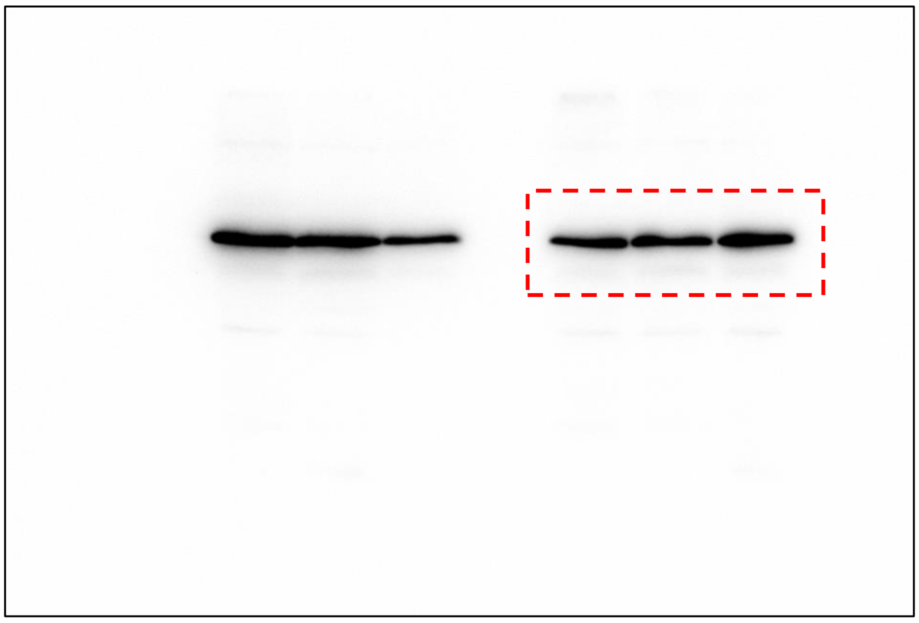

Figure 2d, α-ACTIN blot

Supplementary Information 1a. Full-length gels served as sources for the cropped images in Figure 2d.
Sections bordered by dashed red line were cut and used in the indicated panels of Figure 2.

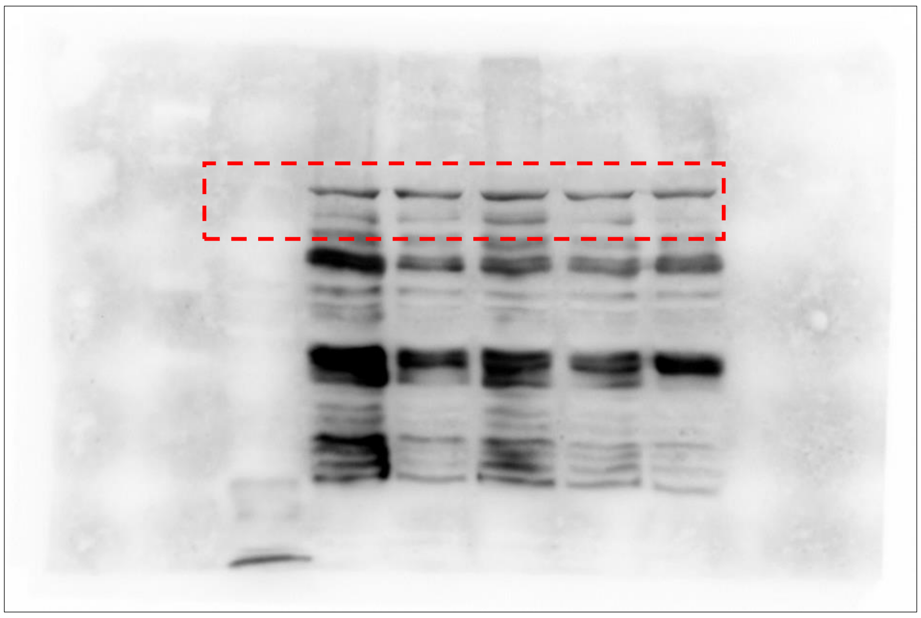

Figure 7a, α-His blot

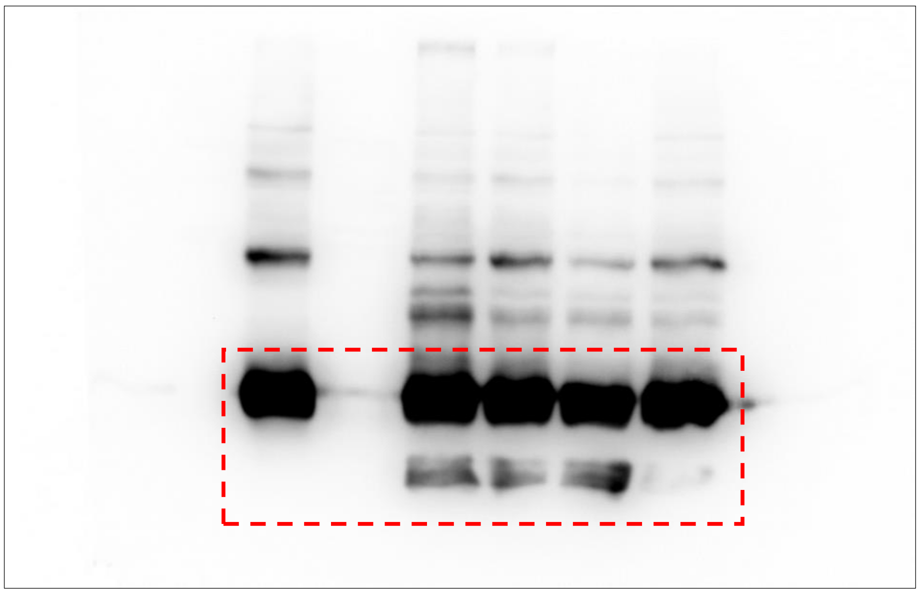

Figure 7a, α-Ub blot

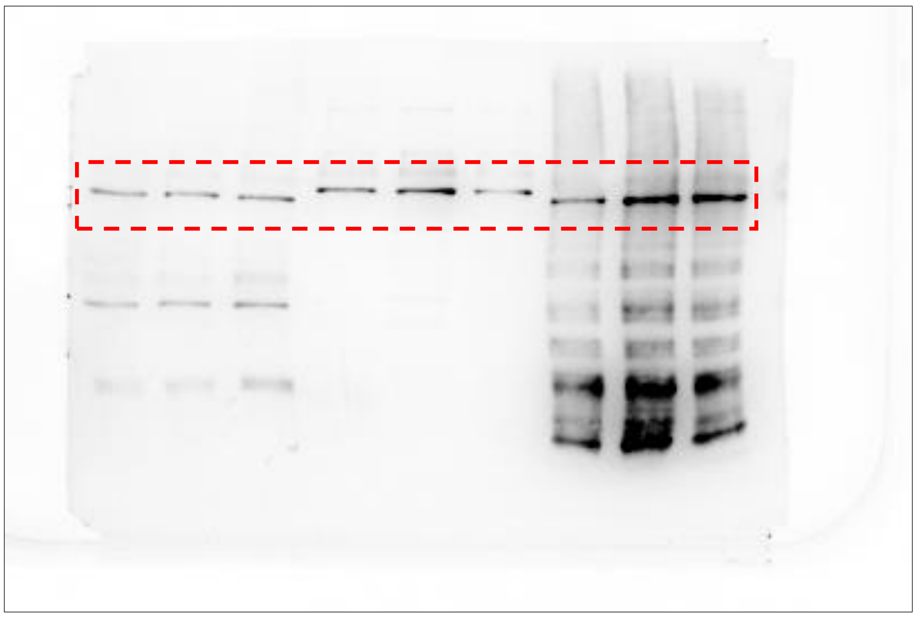

Figure 7b, α-His blot

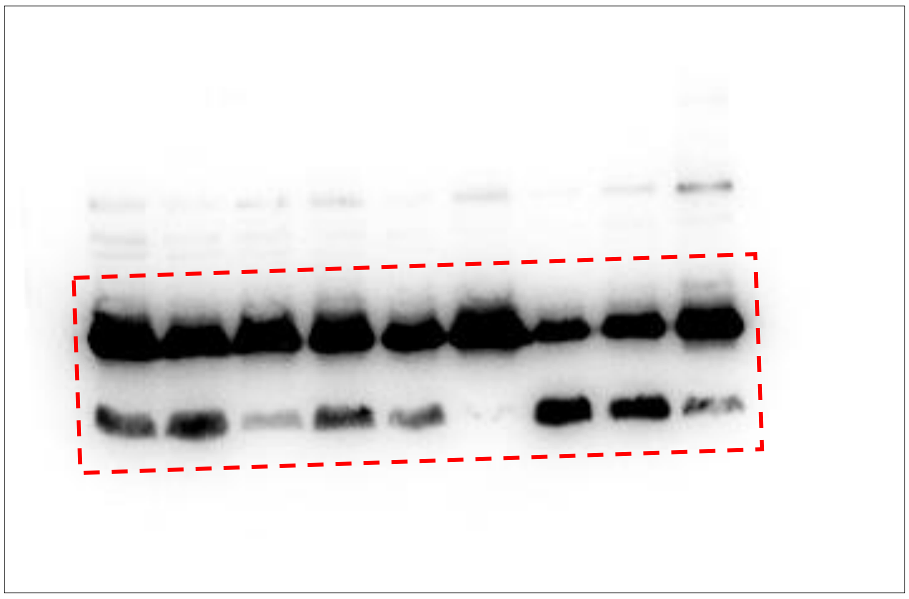

Figure 7b, α-Ub blot

Supplementary Information 1b. Full-length gels served as sources for the cropped images in Figure 7.
Sections bordered by dashed red line were cut and used in the indicated panels of Figure 7.
